# Prone Position and Epidural Anesthesia Without Secured Airway: Prospective Case Study in Infants Undergoing Surgery for Anorectal Malformations

**DOI:** 10.3390/jcm15114001

**Published:** 2026-05-22

**Authors:** Mira Zeilberger, Peter Marhofer, Markus Zadrazil, Philipp Opfermann, Renate Fartacek, Werner Schmid

**Affiliations:** 1 Department of Anesthesia, Critical Care and Pain Medicine, Medical University of Vienna, Waehringer Guertel 18–20, 1090 Vienna, Austria; mira.zeilberger@meduniwien.ac.at (M.Z.);; 2 Department of Pediatric Surgery, Division of Pediatric Surgery, Medical University of Vienna, Waehringer Guertel 18–20, 1090 Vienna, Austria; 3 Department of Special Anesthesia and Pain Therapy, Medical University of Vienna, Waehringer Guertel 18–20, 1090 Vienna, Austria

**Keywords:** general anesthesia, pediatrics, ultrasonography, anal atresia, prone position, epidural anesthesia

## Abstract

**Background and Aims:** This study investigates the feasibility of performing anorectal surgery in neonates in a prone position via epidural anesthesia and sedation without an instrumented airway. **Methods:** Twenty infants, scheduled for surgery for anorectal malformations, were included in this study. The primary endpoint was the success of the anesthesia method without invasive airway manipulation after skin incision, and secondary endpoints were defined as the need for additional opioids or sedation drugs during the perioperative period. The study was approved by the Ethics Committee of the Medical University of Vienna (ref. 1133/2017—approval date 24 August 2017), and was registered in the German Clinical Trial Register (DRKS ID: DRKS00012683, approval date 15 July 2019, updated 30 July 2020). **Results:** The primary outcome parameter could be achieved in 95% of the cases, and 85% of the cases could be managed without additional opioids (secondary outcome parameter). Only one infant (5%) needed endotracheal intubation due to laryngospasm during prone positioning. **Conclusions:** Surgery for infantile anorectal malformations in a prone position is possible with epidural anesthesia and sedation without an instrumented airway.

## 1. Introduction

Anorectal malformations are the most prevalent congenital defects in children, occurring in approximately 1 in 4000 to 5000 births [1]. The standard surgical procedure is based on the initial description by Pena and Devries, where an anatomical situs is re-established via a posterior sagittal anorectoplasty [2,3]. This surgical procedure is usually performed during the first weeks after birth and requires a prone position with an elevated anorectal region. The standard anesthesia procedure is based on general anesthesia with endotracheal intubation and systemic opioid administration, with a high proportion of prolonged postoperative intubation and mechanical ventilation [4].

An ongoing discussion regarding the neurotoxicity of various general anesthetic drugs [5,6], adverse effects of opioids on postoperative respiration [7], and the challenge of optimizing postoperative pain therapy [8] requires considerations of alternative anesthetic methods. Epidural anesthesia with the subsequent avoidance of an instrumented airway appears as a promising technique to reduce the above-described potentially detrimental effects of an opioid-based general anesthesia and endotracheal intubation [9]. A recent study describes the performance of caudal blockade plus endotracheal intubation for anorectal surgery in small babies [10]. However, the real benefit of neuraxial anesthesia in this patient population is the avoidance of invasive airway manipulation. General anesthesia and mechanical ventilation may adversely impact pulmonary function during and beyond the intraoperative phase [11]; consequently, it is advantageous to avoid mechanical ventilation whenever possible.

Previous studies illustrate the feasibility of epidural anesthesia in spontaneously breathing neonates and small babies for inguinal surgery [12], pyloromyotomy [13], subumbilical laparoscopic procedures [14], open transvesical Cohen ureteric reimplantation surgery [15], and intestinal reconstruction [16]. The difference of the present study to the aforementioned surgical procedures is the intraoperative requirement for prone positioning and impaired approach to the airway. Nevertheless, our scientific focus during the last 25 years was the development of a safe and effective management of various surgical procedures via neuraxial anesthesia without an instrumented airway. The success rates of caudal anesthesia under the guidance of ultrasonography are in the range of 95% with a very low rate of complications [12], and therefore, the extension of this technique for surgical procedures with an impaired approach to the pediatric airway is a consequent step in the further development of opioid-free anesthesia with minimization of airway manipulations.

We therefore conducted this prospective observational study in neonates undergoing surgery for anorectal malformations in a prone position under epidural anesthesia and light sedation without an instrumented airway.

## 2. Materials and Methods

### 2.1. Preparations, Enrolling Patients, and Exclusion Criteria

**Figure 1:**
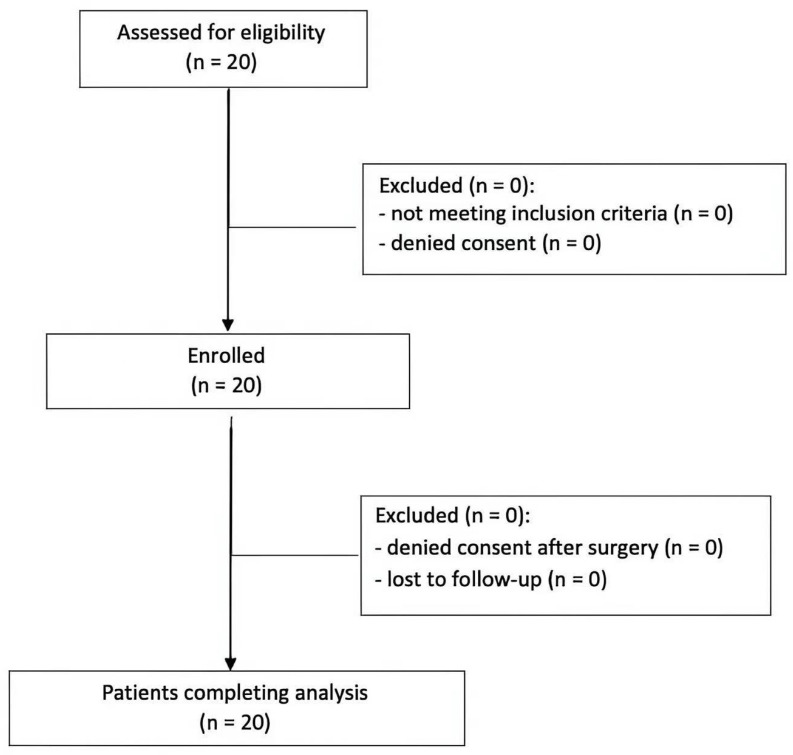
STROBE diagram.

The Ethics Committee at the Medical University of Vienna approved this prospective case series (ref. 1133/2017—approval date 24 August 2017), which was registered in the German Clinical Trial Register (DRKS ID: DRKS00012683, approval date 15 July 2019, updated 30 July 2020), and the study design adhered to the principles outlined in the STROBE statement (Figure 1). The Clinical Trial Register DRKS00012683 indicates a cohort of 80 patients with various surgical indications, where the applicability of epidural anesthesia without a secured airway was defined as the primary endpoint.

The present prospective case series investigates a sub-cohort of 20 neonates and infants scheduled for anorectal surgery in a prone position between 2018 and 2021. We used the number of 20 study participants in previously published case series [14,15,16], and therefore, this quantity appeared appropriate for the selected study design. Children with allergies to aminoamide local anesthetics, coagulopathies or anatomical abnormalities (e.g., spina bifida, meningomyelocele) were excluded from the study. Written consent was obtained from parents or legal guardians of all participating children, who were informed about the study’s nature, scope, and procedures.

### 2.2. Anesthesia Management

After standard fasting guidelines at the time of the study (4 h for breast and formula milk, 2 h for clear fluids), children were transferred from the preoperative area to the operation room, where cardiorespiratory monitoring (ECG, non-invasive arterial pressure, SpO_2_) and a forced-air warming blanket (Bair Hugger^TM^; Arizant, Eden Prairie, MN, USA) were established. Sedation began with a face mask delivering 8 Vol% sevoflurane (Sevorane^TM^, AbbVie, Campoverde di Aprilia, Italy) in 50% oxygen/air, followed by a vascular access and an initial bolus of propofol (Fresenius Kabi, Uppsala, Sweden) in a dose range between 1 and 2 mg kg^−1^. Thereafter, sedation was maintained via a continuous propofol infusion of 5 mg kg^−1^ h^−1^. Fluid management was based on Elo-Paed balanced 10 mL kg^−1^ h^−1^ (Fresenius Kabi, Graz, Austria). During the entire period of induction and maintenance of sedation, spontaneous breathing was maintained. An end-tidal CO_2_ line, connected to a face mask secured with adhesive tape, where an oxygen/air mixture (FiO_2_ 0.40) was delivered, provided confirmation of spontaneous breathing.

### 2.3. Ultrasound-Guided Neuraxial Procedures

All neuraxial regional anesthetic techniques were performed via ultrasound guidance using a high-end mobile ultrasound device with a linear 12–3 MHz ultrasound transducer (SonoSite M-Turbo, SonoSite Inc., Bothell, WA, USA) as previously described [17,18]. After induction of sedation as above described, children were placed on the left lateral side with the knees flexed. According to the surgical plan, caudal or epidural puncture at the thoracolumbar transition (if more proximal colorectal surgery was required) was performed. For caudal punctures, we used the ‘immobile needle technique’ [19] with a short-bevel 24 G canula and a prefilled injection line, injecting 1.0 mL kg^−1^ ropivacaine 3.8 mg mL^−1^ (Sintetica Inc., Muenster, Germany) into the caudal space. For epidural punctures, we used a 20 G Tuohy canula and a loss-of-resistance syringe, injecting 0.5 mL kg^−1^ ropivacaine 3.8 mg mL^−1^ into the epidural space. Sterile preparation of the puncture site, preparation of the ultrasound probe with a sterile ultrasound probe cover (Safersonic^TM^, Persenbeug, Austria) and use of a sterile ultrasound transmission gel (Safersonic^TM^, Persenbeug, Austria) were mandated.

### 2.4. Assessment of Anesthesia, Prone Positioning and Emergency Procedures

Ten minutes after performing the blockade, surgeons conducted Pinprick testing by applying stimulation with forceps to the area of the surgical incision. Successful blockade was defined via unaltered hemodynamic parameters (increase in heart rate and blood pressure < 15% from initial values) and no movement of the infants.

**Figure 2:**
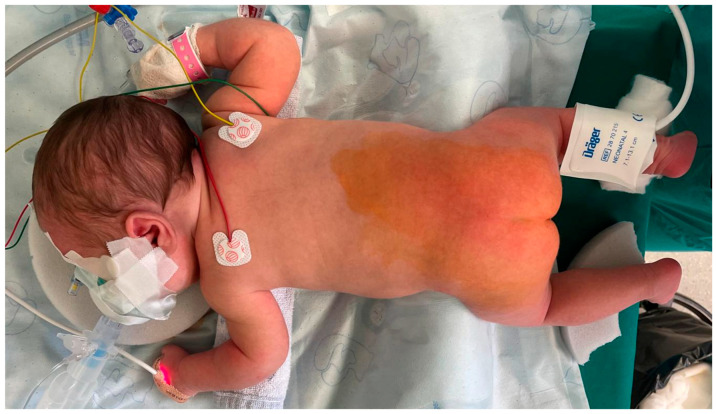
Child in prone position after performance of neuraxial anesthesia in spontaneous respiration prior to surgery.

Thereafter, children were carefully brought into a prone position, and particular care was focused on the position of the head to maintain spontaneous respiration (Figure 2). During surgery, any movement of the legs or increase in hemodynamic parameters ≥ 15% from baseline was considered as inadequate blockade, followed by intravenous administration of fentanyl (Hameln Pharma Inc., Modra, Slovakia) 5 µg kg^−1^ or propofol 1–2 mg kg^−1^. Due to the impeded access to the airway, we were prepared to immediately interrupt surgery in cases of incomplete regional anesthesia blockade and (due to additional pain pharmaceuticals) subsequent brady-/apnoea, turning the child back to a supine position to manage the airway by establishing an endotracheal tube. Propofol 4 mg kg^−1^, rocuronium (Fresenius Kabi Inc., Graz, Austria) 0.6 mg kg^−1^, and fentanyl 5 µg kg^−1^ were administered in those cases prior to endotracheal intubation. Bradycardia or hypotension ≥ 15% below baseline was treated with atropine (Takeda Austria Inc., Linz, Austria) 0.02 mg kg^−1^ or a fluid bolus of 10 mL kg^−1^, respectively.

### 2.5. Postoperative Management in the Recovery Room and Final Examination

After the children were transferred to the post-anesthesia care unit, their pain was assessed with the Face, Legs, Activity, Cry, and Consolability (FLACC) score [20]. This scoring system evaluates observable behaviors like crying, facial expressions, torso and leg posture, and physical restlessness. Each behavior is rated as 0 (none), 1 (moderate), or 2 (severe), yielding a total score up to 10. Assessments occurred both upon arrival in the recovery room and every 30 min during the postoperative period until the neuraxial anesthesia wore off. If a child had a FLACC score of 4 or higher on two consecutive checks, they were administered nalbuphine (AOP Orphan Pharmaceuticals Inc., Vienna, Austria) at a dose of 0.1 mg kg^−1^. Additionally, clinicians examined the puncture site for local infection 24 h after surgery.

### 2.6. Study Endpoints and Data Analysis

The primary endpoint was defined as the successful completion of surgery under regional anesthesia with patients maintaining spontaneous respiration after skin incision. Secondary endpoints were defined as the use of fentanyl or additional propofol during surgery, and the administration of postoperative analgesics in the recovery room.

Data were recorded and analyzed using computerized spreadsheets (Excel 2016; Microsoft, Redmond, WA, USA) and statistical software (Prism 10.4.1; GraphPad Software Inc., San Diego, CA, USA). Because tests for normal distribution lack sufficient statistical power at a sample size of n = 20, we a priori adopted a non-parametric statistical approach. Accordingly, continuous data are expressed as medians [interquartile range] and categorical data as numbers (percentages).

## 3. Results

All eligible patients (n = 20) were included in this study. Patient flow, relevant morphometric/regional anesthesia-related data and surgical indications are presented in Figure 1, Table 1 and Table 2, respectively.

The primary endpoint (successful completion of surgery under regional anesthesia and sedation, with the patient maintaining spontaneous respiration) was achieved in 95% of cases. One child required endotracheal intubation due to the appearance of laryngospasm during positioning.

Three children (15%) received additional opioids/sedatives during the intraoperative period, whereas no child required additional pain drugs during the postoperative period in the recovery room (secondary endpoints).

No child showed a local infection at the puncture site 24 h after surgery.

## 4. Discussion

This is the first study where neonatal surgery in a prone position is performed with regional anesthesia and spontaneous respiration without an instrumented airway. The primary outcome parameter (performance of the surgical procedure without an instrumented airway and without opioids after skin incision) could be achieved in 95% of the cases. One child required endotracheal intubation following the onset of a laryngospasm during prone positioning. Only three infants required additional opioids intraoperatively (secondary outcome parameter).

The already-published standard anesthesia procedure for surgery for anorectal malformations is general anesthesia with endotracheal intubation. Zhang et al. describe in a large retrospective study delayed extubation in 60.2% of those cases, where age group, body weight, intubation history, preoperative hemoglobin and ASA status are considered as possible factors for delayed extubation [4]. Interestingly, all cases with additional use of caudal anesthesia together with endotracheal intubation were not associated with delayed extubation. The authors of this study state in the Discussion Section that “applying region block during surgery can achieve early extubation and ensure patient safety”. Previous studies show that various surgical procedures can be performed in pure regional anesthesia in sedation and without an instrumented airway [12,13,14,15,16]. The possible benefits of these procedures include a significant reduction in perioperative opioid use, decreased exposure to medications that may be associated with neurotoxic risks, and the elimination of the need for invasive airway management. Infants included in this study represent a particularly sensitive patient population. Therefore, whenever possible, surgical procedures should be performed with regional anesthesia without advanced airway management and with minimization of pharmaceuticals that are potentially neuroapoptotic.

The prerequisite for the successful implementation of the investigated anesthesia method is the safe and effective performance of caudal/epidural regional techniques in daily clinical practice. The first description of caudal blockade in children goes back to 1930 [21], where blocks were performed strictly based on anatomical landmarks. Even with the pure landmark-based method, success rates were around 95% [22,23], but the implementation of ultrasound for confirmation of the correct placement of the needle and the local anesthetics increases the first-puncture success rate significantly [24]. A recent retrospective analysis of 2547 children receiving ultrasound-guided caudal blockade, where 323 (13.3%) of them underwent surgery < 46 weeks after conception, showed a failure rate of 4.9% (2.7% in children between 0 and 3 months, 3.3% in children between 4 and 12 months) [14]. It needs to be highlighted that children in this study were operated on in pure caudal blockade, whereas the majority of similar investigations in that field represent data where combined anesthesia techniques were used. Interestingly (but not surprisingly), the younger and smaller the children are, the more respiratory complications are observed [12]. Therefore, the one single respiratory complication requiring endotracheal intubation in the recent study is in accordance with previous data in our smallest patients.

Less robust data are available for epidural anesthesia at the lumbar or thoracolumbar transition level. Bösenberg described this technique in 240 neonates with a high success rate and minimal complications (3/240 = 1.25%) [25]. It was again our study group modifying this regional anesthetic technique with ultrasound [18], but large outcome studies in this field with exact evaluations of success rates and side effects are lacking. Nevertheless, if a higher regional block above the umbilicus is required due to expected surgical indications, ultrasound-guided epidural anesthesia at the lumbar or thoracolumbar transition level can be performed in a safe and effective manner.

Minimization of respiratory support with early extubation in neonates after anorectal surgery is already described by Cui et al. in 12 cases, where general anesthesia is used with endotracheal intubation, but without the use of opioids and muscle-relaxation plus caudal blockade in combination [26]. The authors of this study highlight that “the choice of anesthesia strategy and anesthetic can help to achieve rapid postoperative rehabilitation and save hospital costs”, which is in accordance with the opinion of the authors of the present study. Furthermore, we believe that nowadays expertise in pediatric regional anesthesia enables the avoidance of airway management for many surgical indications, thus realizing the target of minimally invasive and cost-effective anesthesia management.

We present a case series model for this new anesthesiological approach in anorectal surgery. We are aware that case series have less scientific value compared to randomized studies. Nevertheless, previously selected surgical procedures in regional anesthesia plus sedation without an instrumented airway are already successfully described via case series [27], but subsequent studies need to be published to re-evaluate the initial results [13]. Therefore, further studies are also required to re-evaluate the findings of the present study. Selection and performance biases are also important issues in the critical assessment of the current study, which was performed in a highly specialized center. The generalizability of the study results needs to be confirmed in other settings. Nevertheless, a certain specialization in pediatric anesthesia with particular expertise in pediatric regional anesthesia is the prerequisite for performing such cases with a maximum level of safety and efficacy. Finally, the above-mentioned cost-saving effects also need to be confirmed for surgery for anorectal malformations.

While procedural sedation in the prone position with an unprotected airway intuitively raises concerns regarding pulmonary aspiration, the actual incidence of clinically significant aspiration in pediatric anesthesia is exceedingly low. According to the large-scale European APRICOT trial, the overall incidence of bronchial aspiration in children is estimated at 9.3 per 10,000 cases [28], and the event is associated with low morbidity [29]. The single case in our study requiring endotracheal intubation was due to a laryngospasm during positioning. The respiratory event was most likely caused by tactile stimulation during positioning and movement of the head, demonstrating that infants are highly sensitive even when procedures are performed with the utmost care.

The prone position itself does not mitigate the risk of aspiration. It rather introduces specific airway management challenges when airway-related problems become evident during the surgical procedure. The safety of sedation during the prone position with a non-instrumented airway relies heavily on the strict absence of patient-related risk factors, a rigorous adherence to standard preoperative fasting guidelines [30], and the immediate availability of advanced airway management devices, skilled anesthesia personnel, and full preparation for rapid sequence intubation. However, this case series contains an instance in which endotracheal intubation was necessary, underscoring the importance of maintaining diligent attention and vigilance throughout the procedure—even within an experienced clinical environment under controlled study conditions.

In summary, the present study is the first description of neuraxial anesthesia for surgery in the prone position. We describe the application of epidural (caudal or lumbar) anesthesia in sedation without an instrumented airway for anorectal surgery with a 95% success rate where only three infants required additional opioids. Additional research via alternative study concepts (e.g., multicenter observational studies) should be considered to determine whether further outcome measures—such as long-term postoperative analgesia requirements or rapid initiation of enteral feeding—can be optimized through this anesthesia technique. Performing these procedures safely and effectively demands advanced manual skills and a high level of vigilance from anesthesiologists, yet patients benefit from not needing airway management.

## 5. Conclusions

This study is another example of the application of regional anesthesia in complex pediatric surgery. Airway management in this patient population is still associated with a notable morbidity, and therefore, the combination of regional anesthesia plus sedation without an instrumented airway appears advantageous. Invasive neonatal surgery in the prone position in neuraxial anesthesia without endotracheal intubation was not described until today. The presented 20 cases, which were managed as described in this manuscript, may serve as a starting point for further investigations in this field.

## Figures and Tables

**Table 1:** Morphometric and regional anesthesia/additional pain management-related data.

			n
Children included in the study			20
Age at the time of surgery (days)	54	[14–173]	
Preterm babies (<37 weeks postgestational week)			3 (15)
Male/Female			14/6
Weight (kg)	4.7	[3.6–6.9]	
Height (cm)	55	[52–64]	
Duration of surgery (min)	75	[60–111]	
Total volume of ropivacaine for caudal blockade (mL)	5.0	[3.9–5.9]	16
Total volume of ropivacaine for epidural anesthesia (mL)	3.5	[2.6–5.8]	4
Cases that could be managed opioid-free			17 (85)
Fentanyl (µg)	15	[5–49]	3 (15)

Data are median values [interquartile range] or numbers (percentages).

**Table 2:** Indications for surgery.

	n
Anorectal malformation with perianal fistula	6
Anorectal malformation without fistula	5
Anorectal malformation with recto-prostatic fistula	4
Anorectal malformation with recto-scrotal fistula	2
Anorectal malformation with vestibular fistula	1
Anorectal malformation with recto-urethral fistula	1
Anorectal malformation with recto-vestibular fistula	1

## Data Availability

The data relevant to this study cannot be made publicly available, as it contains sensitive patient information concerning a vulnerable population (infants under 1 year of age). Access to the data is restricted by the Data Access Committee of the Medical University of Vienna in accordance with Article 9 of the General Data Protection Regulation (GDPR). For inquiries or data access requests, please contact: datenclearing@meduniwien.ac.at “https://www.meduniwien.ac.at/web/en/about-us/organisation/committees/data-clearing-house/ (accessed on 2 April 2026)”.

## References

[1] Herman R.S., Teitelbaum D.H. (2012). Anorectal malformations. Clin. Perinatol..

[2] de Vries P.A., Peña A. (1982). Posterior sagittal anorectoplasty. J. Pediatr. Surg..

[3] Peña A., Devries P.A. (1982). Posterior sagittal anorectoplasty: Important technical considerations and new applications. J. Pediatr. Surg..

[4] Zhang Q., Xu J., Huang Q., Gong T., Li J., Cui Y. (2024). Risk factors for delayed extubation after pediatric perineal anaplasty in patients less than 1 year of age: A retrospective study. BMC Pediatr..

[5] Creeley C.E. (2016). From Drug-Induced Developmental Neuroapoptosis to Pediatric Anesthetic Neurotoxicity-Where Are We Now?. Brain Sci..

[6] McCann M.E., Soriano S.G. (2012). General anesthetics in pediatric anesthesia: Influences on the developing brain. Curr. Drug Targets.

[7] Hamilton A.R.L., Yuki K., Fynn-Thompson F., DiNardo J.A., Odegard K.C. (2025). Perioperative Outcomes in Congenital Heart Disease: A Review of Clinical Factors Associated with Prolonged Ventilation and Length of Stay in Four Common CHD Operations. J. Cardiothorac. Vasc. Anesth..

[8] Kinoshita M., Borges do Nascimento I.J., Styrmisdóttir L., Bruschettini M. (2023). Systemic opioid regimens for postoperative pain in neonates. Cochrane Database Syst. Rev..

[9] Marhofer P., Lönnqvist P.-A. (2014). The use of ultrasound-guided regional anaesthetic techniques in neonates and young infants. Acta Anaesthesiol. Scand..

[10] Sultanova M.C., Nasibova E.M. (2022). Caudal anesthesia as an alternative for the correction of anorectal defects in children. Pediatr. Anesth. Crit. Care J..

[11] Trachsel D., Svendsen J., Erb T.O., von Ungern-Sternberg B.S. (2016). Effects of anaesthesia on paediatric lung function. Br. J. Anaesth..

[12] Opfermann P., Kraft F., Obradovic M., Zadrazil M., Schmid W., Marhofer P. (2022). Ultrasound-guided caudal blockade and sedation for paediatric surgery: A retrospective cohort study. Anaesthesia.

[13] Opfermann P., Wiener C., Schmid W., Zadrazil M., Metzelder M., Kimberger O., Marhofer P. (2021). Epidural versus general anesthesia for open pyloromyotomy in infants: A retrospective observational study. Paediatr. Anaesth..

[14] Opfermann P., Marhofer P., Springer A., Metzelder M., Zadrazil M., Schmid W. (2022). A prospective observational study on the feasibility of subumbilical laparoscopic procedures under epidural anesthesia in sedated spontaneously breathing infants with a natural airway. Paediatr. Anaesth..

[15] Opfermann P., Zadrazil M., Tonnhofer U., Metzelder M., Marhofer P., Schmid W. (2022). Ultrasound-guided epidural anesthesia and sedation for open transvesical Cohen ureteric reimplantation surgery in 20 consecutive children: A prospective case series and proof-of-concept study. Minerva Anestesiol..

[16] Marhofer D., Zadrazil M., Opfermann P.L., Wiener C., Marhofer P., Schmid W. (2025). Intestinal Reconstruction in Infants Under Epidural Anesthesia Without Invasive Airway: A Prospective Case Study. J. Clin. Med..

[17] Wiegele M., Marhofer P., Lönnqvist P.-A. (2019). Caudal epidural blocks in paediatric patients: A review and practical considerations. Br. J. Anaesth..

[18] Willschke H., Bosenberg A., Marhofer P., Willschke J., Schwindt J., Weintraud M., Kapral S., Kettner S. (2007). Epidural catheter placement in neonates: Sonoanatomy and feasibility of ultrasonographic guidance in term and preterm neonates. Reg. Anesth. Pain Med..

[19] Kumagai M., Yamashita M. (1995). Sacral intervertebral approach for epidural anaesthesia in infants and children: Application of “drip and tube” method. Anaesth. Intensive Care.

[20] Merkel S.I., Voepel-Lewis T., Shayevitz J.R., Malviya S. (1997). The FLACC: A behavioral scale for scoring postoperative pain in young children. Pediatr. Nurs..

[21] Martin J.G. (1930). Caudal Block. Ind. Med. Gaz..

[22] Dalens B., Hasnaoui A. (1989). Caudal anesthesia in pediatric surgery: Success rate and adverse effects in 750 consecutive patients. Anesth. Analg..

[23] Talwar V., Tyagi R., Mullick P., Gogia A.R. (2006). Comparison of ‘whoosh’ and modified ‘swoosh’ test for identification of the caudal epidural space in children. Paediatr. Anaesth..

[24] Jain D., Hussain S.Y., Ayub A. (2022). Comparative evaluation of landmark technique and ultrasound-guided caudal epidural injection in pediatric population: A systematic review and meta-analysis. Paediatr. Anaesth..

[25] Bösenberg A.T. (1998). Epidural analgesia for major neonatal surgery. Paediatr. Anaesth..

[26] Cui Y., Wang Y., Cao R., Liu K., Huang Q.-H., Liu B. (2019). On-table extubation in neonates undergoing anoplasty: An experience of anesthetic management on the concept of fast-tracking anesthesia: A pilot study. Medicine.

[27] Willschke H., Machata A.M., Rebhandl W., Benkoe T., Kettner S.C., Brenner L., Marhofer P. (2011). Management of hypertrophic pylorus stenosis with ultrasound guided single shot epidural anaesthesia--a retrospective analysis of 20 cases. Paediatr. Anaesth..

[28] Habre W., Disma N., Virag K., Becke K., Hansen T.G., Jöhr M., Leva B., Morton N.S., Vermeulen P.M., Zielinska M. (2017). Incidence of severe critical events in paediatric anaesthesia (APRICOT): A prospective multicentre observational study in 261 hospitals in Europe. Lancet Respir. Med..

[29] Kelly C.J., Walker R.W. (2015). Perioperative pulmonary aspiration is infrequent and low risk in pediatric anesthetic practice. Paediatr. Anaesth..

[30] Green S.M., Leroy P.L., Roback M.G., Irwin M.G., Andolfatto G., Babl F.E., Barbi E., Costa L.R., Absalom A., Carlson D.W. (2020). Fasting before procedural sedation: A consensus statement. Anaesthesia.

